# The needs and experiences of critically ill patients and family members in intensive care unit of a tertiary hospital in Malaysia: a qualitative study

**DOI:** 10.1186/s12913-023-09660-9

**Published:** 2023-06-13

**Authors:** E-Li Leong, Chii-Chii Chew, Ju-Ying Ang, Sharon-Linus Lojikip, Philip-Rajan Devesahayam, Kit-Weng Foong

**Affiliations:** 1 Clinical Research Centre, Hospital Raja Permaisuri Bainun, Ministry of Health, 30450 Ipoh, Perak Malaysia; 2 Otolaryngology Department, Hospital Raja Permaisuri Bainun, Ministry of Health, Ipoh, Malaysia; 3 Anaesthesiology and Intensive Care Department, Hospital Raja Permaisuri Bainun, Ministry of Health, Ipoh, Malaysia

**Keywords:** Needs, Experiences, Critically ill patients, Family members, Intensive care unit

## Abstract

**Background:**

Admission to an intensive care unit (ICU) is a stressful experience for patients and their family members. While the focus of management is primarily on medical care, there can be other areas which are overlooked. The purpose of this study was to investigate the needs and experiences of ICU patients and family members.

**Method:**

This qualitative study involved four trained researchers conducting in-depth interviews (IDI) based on a semi-structured interview guide. The participants were ICU patients and family members. All IDIs were audio-recorded and transcribed verbatim. Four researchers independently analyzed the data via thematic analysis with the aid of QDA Miner Lite®. The themes and subthemes were generated and confirmed by literature and expert opinion.

**Results:**

Six IDIs were conducted with three patients and three family members, whose ages ranged from 31 to 64 years old. One pair of participants consisted of a patient and his respective family member, while the other four participants did not have a familial relationship with each other. Three main themes emerged from the analysis: (I) critical care services; (II) physical spaces; and (III) monitoring technology. Medical, psychological, physical, and social needs for critical care services were expressed by both patients and family members. Patients’ needs in clinical spaces were highlighted as a conducive ICU environment with ambient temperature and controlled noise levels. In non-clinical spaces, family members expressed a need for more chairs in the waiting area. Participants expressed the need for call bells as well as patients’ negative perceptions of medical equipment alarms in the ICU when it pertained to monitoring technology.

**Conclusion:**

This study provides an in-depth view at the needs and experiences of ICU patients and family members who have a variety of unmet needs. This understanding is critical for guiding ICU personnel and stakeholders in their efforts to humanize ICU care.

## Background

Critical care is a multidisciplinary and inter-professional specialty that manages patients with acute, and life-threatening organ dysfunction. Admission to the intensive care unit (ICU) causes distress to both patients and their families [1]. ICU patient care emphasizes a holistic approach that not merely focuses on medical care but also aims to fulfill patients’ and families’ needs [2, 3]. The needs identified by the patients warded in ICUs across different countries are broadly categorized as physical, medical, psychological, and social needs [4]. Generally, ICU patients desire individualized care from the medical personnel and prompt medical attention when the need arises. The patients also need to understand their medical conditions to make decisions on treatment priorities and at the same time to alleviate fear, anxiety, and panic attacks. A secure environment that creates a sense of security, fosters self-worth and motivation toward recovery is essential [5–8].

A sense of hope is an important need for family members of patients who are admitted to the ICU. Additionally, they require reassurance from the ICU personnel that the care provided is in the patient’s best interest [1]. Having an adequate understanding and attempting to meet the families’ needs would improve their ability to cope with the ICU admissions of their loved ones [9]. There is a slight geographical variation in family member’s needs. In Hong Kong and Malaysia, family members needed assurance that the patients were adequately cared for [10–12]. The primary concerns in Saudi Arabia were information, reassurance, spiritual healing, and support [13]. Moving west, in the United Kingdom, the needs were access to maintain proximity to patients; a positive and supportive environment; information; and hope [14]. An open communication and regular updates build mutual trust between the ICU personnel and the family members. In Ireland, families expressed their needs for truthful updates on the patient’s condition; understanding ICU admission is a dynamic and continuous process; being with their relatives; having the nurses’ assurances; and support for coping [15]. The difference in priorities and needs globally reflect disparities in culture, religion, and healthcare. In Malaysia, the challenge in providing culturally sensitive intensive care is further compounded due to its multiracial composition with diverse cultural and religious backgrounds.

Patient experience is defined as interactions that patients have with the healthcare system, including their management plan, the providers, and the providers’ practices in the healthcare institution [16]. Their experience in the ICU affects them physically and psychologically. The common physical discomforts experienced include pain, sleepiness, discomfort, inactivity or over-activity, noise, thirst, headache, discomfort associated with endotracheal tubes, and swallowing difficulties. Psychologically, they are affected by their disease progression, medical treatment, and perception of care concerning the manner and behavior of the ICU personnel. Patients reported hallucinations, fear, worry, anxiety, melancholy, loneliness, death thoughts, panic, uneasiness, uncertainty and despair [17]. These areas of patient experience can be easily overlooked when their critical illnesses are the primary focus of the managing team.

Family members experience distress when they learn that their loved ones have been admitted to the ICU. Emotional helplessness is experienced by family members when their need for information, reassurance, help, and support have not been met. They experience a lack of control, uncertainty, and loneliness. They undergo intense emotional changes whenever there is a morbid change in the status of their relatives. Family members are frustrated when their experiences deviate from their expectations, which are affected by their cultural background [18]. Gauging the needs and experiences of patients and their family members is a crucial step forward in establishing humanized ICU care. This study aims to learn about the needs and experiences of critically ill patients and their family members in a local setting.

## Methods

### Study design and setting

This exploratory qualitative study was conducted from February to March 2020 in a multidisciplinary ICU of a tertiary government hospital in Malaysia. The ICU had two wards with a total of 23 beds, with 1200 to 1400 admissions per year. A multi-disciplinary team of 150 to 160 personnel in the ICU includes two consultant intensivists, ICU trainees, anaesthesiologists, anaesthesia-trained medical officers, staff nurses, physiotherapists, pharmacists and attendants. The staff nurses in the ICU worked in shifts, and the medical doctors were subject to an on-call arrangement. The patients’ families were permitted to visit them twice a day, one at a time, between 1 pm and 2 pm and again between 5 pm and 6.30 pm. Due to the lack of a waiting room, family members were compelled to wait in the corridor outside the ICU during visiting hours. The corridor was furnished with seats for ten to fifteen individuals. During non-visiting hours, family members were called by phone if medical doctors wished to discuss the patient’s medical condition with family members. Family conferences were often held in the nursing manager’s office or the Intensivist’s office, while daily patient updates to family members were typically delivered at the bedside.

### Sample and recruitment

#### Inclusion criteria

Patients who were 18 and above, Malaysian, able to read and speak English, Malay or Mandarin, had their first ICU encounter, admitted to the ICU for at least 72 h to make sure there was enough time to establish their needs [11, 19], had a Glasgow Coma Scale (GCS) of 15, a Richmond Agitation-Sedation Scale (RASS) of 0, a negative Confusion Assessment Method for the ICU (CAM-ICU), and an overall stable health condition at the discretion of consultant intensivists, were fit to participate in a 60-minute interview. Patients were recruited based on different characteristics such as age, gender, and the types of specialty care they received in order to enrich the data.

Family members, not limited to first-degree relatives, who had been the main person interacting with critical care personnel and were willing to share their experience were invited for interviews. The patients and the family members invited to the study were not necessarily related or paired as certain patients may not fit the eligibility criteria to participate in this study while their family members were.

#### Exclusion criteria

Patients with language or communication barriers, with underlying psychiatric disorders or newly diagnosed psychiatric disorders during ICU admission and those with intellectual impairment were excluded. Family members of critically ill patients with unstable vital signs or whose death was considered imminent were not included in this study out of respect for the grieving needs of the family members [19].

### Interviewers

There were a total of four interviewers (ELL, SLL, CCC and JYA), all of whom were trained in qualitative research. Two of the interviewers held graduate degrees in medicine, while the other two held graduate degrees in pharmacy. Prior to the interview, the interviewers did not know the participants nor did they establish a relationship with them. Interviewers were guided by a semi-structured interview guide, which ensured consistency in the domains covered during each IDI. Additionally, prior to the actual data collection, trial interviews were conducted among the interviewers. These trial interviews served the purpose of establishing a shared understanding of the interview guide and techniques, thereby ensuring a more uniform approach across the IDIs.

### Sample size and sampling method

Patients and their family members were recruited through purposive sampling. The potential patients were identified during daily ward rounds, and the family members were identified via prior interaction for patients’ updates by the treating intensivist. The participants who agreed to participate were then referred to the interviewers for a scheduled face-to-face interview.

### Instrument

A semi-structured interview guide in English was created separately for patients and family members based on existing literature that reported patients’ and families’ needs in ICU [20–23], as well as expert opinion. The domains were perception of the ICU, the experience of interacting with the health care personnel working in the ICU, information required, perspectives on medical care, the need for privacy (only applicable for patients), types of support needed, and requirements of facilities in the ICU. Subsequently, these guides were translated into Malay and Mandarin by native speakers of Malay and Chinese. Each language’s interview guide was pre-tested to ensure the comprehensibility of terms and phrases used in the interview guide.

### Data collection

Approval to conduct this study was obtained from the Medical Research and Ethics Committee, Ministry of Health Malaysia with the protocol number NMRR-19-3358-51827 (IIR) prior to data collection.

Each participant signed a written informed consent form before data collection. The sociodemographic information of the participants was collected prior to the IDIs. The IDI sessions with the patients were conducted at the bedside, with curtains or blinds drawn to provide privacy. IDI sessions for family members were held in a private office room inside the ICU. Only two interviewers and a participant were present during each IDI. The IDIs lasted 40 to 60 min and were all audio-recorded. There were no additional interviews conducted. This study was terminated after the sixth IDI due to the prohibition on visitors and researchers entering the ICU, as well as possible changes in participants’ perspectives during the COVID-19 pandemic. All IDIs in this study were conducted before the implementation of pandemic-related movement restrictions in Malaysia [24]. Despite this, the last two consecutive IDIs did not yield any new themes, indicating that data saturation had been reached.

### Data analysis

Three patients and three family members participated in this study. Coincidentally, there was only one pair of patient-family members who took part in this study. The remaining four participants were not patient-family members paired. The data collected from the patient-family member pair were analyzed separately.

The audio recordings were transcribed verbatim and the transcripts were not returned to the participants for verification. Data management was conducted using QDA Miner Lite®, and data analysis was performed following the six steps of thematic analysis established by Braun and Clark [25]. All researchers (ELL, SLL, CCC, and JYA) familiarized themselves with the transcript, and each transcript was independently coded by two researchers, with any disputes of coding being resolved by discussion and consensus between researchers. Emerging themes were later categorized based on the five domains of care for critically ill patients, reported by the World Federation of Societies of Intensive and Critical Care Medicine. The domains are (I) critical care services, (II) physical space, (III) monitoring technology, (IV) human resources, and (V) research and quality improvement [26]. Relevant sub-themes were grouped under respective themes. Study findings were further validated by the literature and expert opinion. Non-English quotes were translated into English by one researcher and were cross-checked by another researcher to ensure the accuracy of the translation.

## Results

The participants’ age ranged from 31 to 64 years old. The patients’ median age was 42 (IQR = 15.5) while the family members’ median age was 60 (IQR = 14.5). There were three Malay and three Chinese. Four of them were females and were all married. Four of the six participants had completed secondary school, one had completed primary school, and the other had completed university education. ICU stays ranged from 6 to 182 days (Table 1).

**Table 1:** Characteristics of participants (*n* = 6)

Participants	Discipline	Duration of ICU stay	Relationship with patient
Patient #1	Medical	80	N/A
Patient #2	Medical	10	N/A
Patient #3	Otolaryngology	6	N/A
Family member #1	Medical	182	Spouse
Family member #2	Neurosurgical	48	Spouse
Family member #3	Medical	10	Spouse

*N/A *not applicable

A total of three themes emerged in this study: (I) critical care services, (II) physical space, (III) monitoring technology; with several subthemes identified under each of them (Table 2).

**Table 2:** Themes, subthemes and sub-subthemes of critically ill patients’ and family members’ needs and experience in ICU

Themes	Sub-themes	Sub-subthemes
**Critical care services**	• Medical needs	• Continuous pain relief management • Effective communication in the ICU • Cultural competence care • Participation in patient care • Decision making
	• Psychological needs	• ICU personnel support • Religious support • Patient counselling
	• Physical needs	• Comfortable bed-bath
	• Social needs	• Longer visiting hours
**Physical spaces**	• Clinical spaces	• Conducive ward
	• Non-clinical spaces	• Conducive waiting area
**Monitoring technology**	• Perceptions of medical equipment alarms	
	• Call bells	

### Theme 1: critical care services

Critical care services needs and what the participants had experienced were not limited to the immediate need for the treatment of individual patients, but also the services that extend beyond basic care. The patients’ and family members’ need for critical services were further classified into medical, physical, psychological and social needs.

#### Medical needs

The medical needs of patients and their family members were identified based on their experience during the patients’ ICU stay. The needs include continuous pain relief management, effective ICU communication, a decision-making process, the provision of continuity of care and culturally competent care.

##### Continuous pain relief management

Pain experience was one of the concerns of ICU patients, the continuous need for pain relief has been reiterated by the patients in this study. Necessary analgesics that could not be provided on time have been a concern of ICU patients.

*… if I need the medication to alleviate toothache, he (staff) could not give [the pain medication] immediately, [he will] delay in giving [the pain medication].*

(Patient #3)

##### Effective communication in the ICU

This is part of the essential medical need that occurred between “patient-critical care personnel,“ “family members-patient” and “family members-critical care personnel”. “Patient-critical care personnel”: The patients described that they were unable to communicate their needs to the critical care personnel due to endotracheal tube barriers.

*Like that time when I was inserted with the [breathing] tube, [and] my urine catheter was blocked, I was unable to call people [for help].*

(Patient #3)

“Family member-patient”: the family members had difficulties learning about the needs of the intubated patients.

*We (family members) could not guess what he (intubated patient) was saying.*

(Family member #2)

“Family members-critical care personnel”: Ineffective communication was seen in the non-synchronized conveying of patient information among ICU personnel. Family members were confused by the disparities in the information provided by different ICU personnel.

*When I came in [to ICU], I told [the nurse that] Dr. XXX allowed me to come in [to see my wife] (family member presumed wife’s condition worsened)… I was taken aback (when the nurse responded that), “No, her condition is improving. Why do you want to come (for a visit)?“*

(Family member #1)

Besides, a lack of designated communication channels in the ICU by having specific personnel and allocation of a specific time that allows the family members to get patients’ updates has been raised. The family members were uncertain about who and how to obtain patients’ information updates.

*We could not find a suitable person to ask [regarding patients’ condition], [and] we do not know who to ask [for patients’ condition].*

(Family member #2)

While some family members attempted to obtain information from the nurse, they were instructed to meet the doctors for updates. In contrast, doctors were perceived as rarely seen in the ward during family visiting hours, making it difficult for family members to obtain information.

*But initially, we could not differentiate between who was a doctor and who was a nurse. Sometimes when I asked the nurse, she would say, “you [have to] wait for [the] doctor.” But we hardly saw the doctor when we were here [in ICU during family visiting hours].*

(Family member #2)

Owing to the difficulty in locating the person in charge, a form of communication channel was suggested. The family members preferred bedside name tags that identified the person in charge of a specific patient, allowing them to directly request patient information from that individual.

*Unless the name [of staff in charge] is stated, [then] I will find the person [directly]. That is [one of the] possible [solutions].*

(Family member #3)

##### Cultural competence care

The ability of ICU personnel to provide culturally competent care to patients from diverse backgrounds that take into account language, communication styles, beliefs, attitudes, values, and behavioural diversity was identified as a need in critical care services [27]. The inadequacy of providing cultural competence care was recognised as an issue where some patients encounter language barriers when seeking medical care, necessitating the search for native-speaking critical care personnel to communicate their needs.

*[About] Communication… because I am not very fluent in Bahasa Melayu (Malay language) … So when I see [a] Chinese nurse, I will ask her to help to translate.*

(Patient #2)

Cultural competent care has not been confined to medical care; addressing the beliefs of family members in terms of patients’ nutrition intake was notably a need. A few family members were unsatisfied with the food provided by the hospital to the patients, believing it to be less nutritious for patients.

*Yes, sometimes I see one piece of chicken and some porridge with some squash, [which is] not suitable [for patients].*

(Family member #2)

##### Participation in patient care

This study revealed that family members were willing to learn and perform simple care for their loved ones in the ICU. A family member articulated her willingness to acquire basic skills from the healthcare providers in order to ensure the sustenance of patient care.

*Then, my son-in-law (who is a healthcare professional) taught me something easier, like how to help him (patient) to do phlegm suction (…) so when we see him (visit the patient in ICU), we will do [phlegm suction] by ourselves.*

(Family Member #2)

##### Decision making

The lack of medical treatment knowledge among the participants made them follow the decision of the medical doctors to receive critical care for the patients.

*[We] listen to the doctors regarding all [medical treatment]. We totally have no idea [on medical treatment].*

(Family member #2)

#### Psychological needs

The principal psychological needs in the ICU evolve around the elements, making them feel safe in the ICU. The elements include “knowing”, “hoping”, “trusting” and “regaining control”. These elements are greatly influenced by family and friends, ICU personnel, and religion [28]. This study identified that ICU personnel and religious support were highlighted by the family members as perceived psychological needs of the patients. Patient counselling service was mentioned as an important psychological need for patients in the ICU.

##### ICU personnel support

The ICU personnel is regarded as crucial in terms of providing patients with support and encouragement to live [28]. Some family members believed that the doctors’ encouragement would be more effective in motivating the patients than the families themselves.

*[When] Dr. XXX passed by, he will encourage him (patient) [by saying]: “Uncle [you have] improved a lot over these few days.”*

(Family member #2)

The impact of ICU personnel on the patients’ psychological needs is perceived as substantial, including in a negative way. The family members believed that negative words would harm the patients. Therefore, they requested the doctor in charge to be cautious when disclosing information to patients.

*Doctors’ words (information on patient’s condition) will affect patients (…) Hope [that we] can know [about the patient’s condition], but [we preferred that the doctor] do not disclose [the negative information] in front of the patient.*

(Family member #2)

##### Religious support

Some of the patients expressed a need for religious support while in the ICU.

*Maybe listening to the radio. My husband helped on the radio and played verses from Al-Quran.*

(Patient #1)

##### Patient counselling

The family members agreed that patient counselling service would be one of the best ways to support the patients psychologically.

*It is even better to counsel the patient because the patients need encouragement.*

(Family member #3)

#### Physical needs

##### Comfortable bed-bath

Some of the patients had negative experiences with bed-bath by having to take late-night showers in cold water. There was a need to use warm water for bathing among the ICU patients.

*[It was] eleven (o’clock at night) that [the staff] helped me to shower using … cold water. How [could I] bear with this (showering at night with cold water)? I told them (the staff) that … I would like to use warm water [instead]. He (the staff) agreed [to bring warm water], but what he brought over was cold water. He (staff) told me that … he (staff) would help me to shower at a faster speed. That time I was suffering.*

(Patient #2)

#### Social needs

##### Longer visiting hours

The patients needed longer visiting hours to meet each of their loved ones during hospitalization in the ICU.

*Because they (family members) only [had] one hour [of visiting time]. If it can be extended, [then] we (patient and family member) can talk [longer]… A lot of people (visitors) come [to visit me], [I have] not get to talk to each visitor (within the visiting hour) … So [it will be good] to have longer visiting hours.*

(Patient #2)

### Theme 2: physical spaces

The physical spaces in the ICU are divided into clinical and non-clinical spaces. The clinical space includes the presence of a discrete location where it accommodates the beds, devices, and rooms; a nursing station; and multiple computer stations that are essential in patient care. The pantry, a room for medical personnel to rest, seminar rooms close by, and a place for families to wait are all part of the non-clinical space, which is outside of the physical boundaries of the patient care area [29]. The experience of physical space in the clinical area was described by the patients as a cold and noisy environment. Unmet needs for non-clinical spaces included a lack of visitor chairs in the waiting area.

#### Clinical spaces

##### Conducive ward

Some patients complained of the cold environment in the ICU which resulted in the need to cover themselves with thick layers of blankets.

*The air conditioner was too cold. I needed to cover three layers of blankets, yet [I was] still feeling cold.*

(Patient #3)

Family members were concerned about patients who were unable to sleep at night owing to the noisy environment in the ICU.

*My husband (patient) complained to me that there were a few nights he could not sleep because they (the staff) kept talking loudly.*

(Family member #3)

#### Non-clinical spaces

##### Conducive waiting area

Meanwhile, the participants raised the issue of insufficient chairs for family members in the waiting area, suggesting that the situation could be improved by adding a few additional chairs, especially for elderly visitors.

*I thought that a few more chairs could be added outside the ICU because there were too many people (visitors). Sometimes, some elderly [visitors] do not have chairs to sit on and they need to climb up [the stairs to reach ICU]; yet there was only one row of chairs … I thought it (ICU) should have been equipped with few more chairs.*

(Family member #3)

### Theme 3: monitoring technology

One of the aspects that distinguish critical care from traditional hospital treatment would be the availability of devices with advanced technologies that provide continuous monitoring of a patient’s physiologic status in an ICU [29]. The monitoring technology identified by the participants could be classified into medical devices and non-medical devices. The medical devices in the ICU frequently emitted alarms that startled the patients. On the other hand, the family members were concerned about malfunctioning call bells, which is an important non-medical device.

#### Perception of medical equipment alarms

The ICU was thought to be well-equipped with a variety of medical devices by the majority of the participants. Some were unconcerned about the devices that were attached, while others perceived a sense of hopelessness and fear hearing the alarms and seeing the lights emitted by the medical devices.

*All sorts of sounds (from the devices), [it was] scary (…) [I felt] like no hope [in the ICU].*

(Patient #1)

#### Call bells

One of the family members believed it was dangerous to leave a patient in an isolated room without a functioning emergency call bell, especially if the patient has a health condition that causes breathing difficulties.

*Yes, [you are] right. Sometimes they may be in danger, such as having breathing problems or any other condition, [it is] better to have the bell for them to press, otherwise it is very dangerous [in such a situation].*

(Family member #3)

## Discussions and recommendations

This study uncovered the needs and experiences of critically ill patients and their families in the ICU concerning the critical care services, physical environment, and equipment in the ICU. While previous local studies have focused on the family members of ICU patients [11, 12], this qualitative study in Malaysia investigates the needs and experiences of critically ill patients as well as their family members.

The main medical need that is deemed unmet for the ICU patients in this study would be insufficient pain treatment. This inadequacy was attributed to difficulties in assessing and accurately locating pain, as well as poor awareness among healthcare personnel, particularly among patients who did not undergo any surgical procedures [30]. However, a culture of addressing pain in critically ill patients should be fostered, as dealing with pain complaints promptly has been shown to result in a less stressful stay [17, 31]. One step forward could be reinforcing adherence to the appropriate pain management strategy supported by the Malaysia Ministry of Health [32].

Effective communication in the ICU is reiterated as an essential need of patients and family members. Critically ill patients who received invasive mechanical ventilation experienced communication difficulties. At the same time, ICU personnel and families reported difficulties in understanding patients’ needs as well [33, 34]. The utilization of aided or unaided augmentative and alternative communication systems could assist in meeting this demand, and this should be implemented according to the local culture and setting [35].

The family members in this study had a great desire for up-to-date patients’ information, as identified as a fundamental need of family members [9]. They needed to know about the patients’ progress and prognosis, why certain activities were conducted for the patients, and who to call when they were away [36, 37]. In Malaysia, patients’ information is disclosed by medical doctors and reinforced by nurses to the family members [38]. However, the medical doctors in the ICU tend to prioritise patients’ care, which may result in insufficient time for communication or difficulty locating them when they are managing other patients [34]. Hence, to establish family-centered care in the ICU and obtain standardised patient information promptly, a consensus for a point person, frequency and types of contact should be made between ICU personnel and family members [39]. However, these proposals should be addressed further among the ICU team in order to better adapt to the local system.

Family members’ participation in various patient care activities has been reported, ranging from massage, bathing, eye and mouth care, to positioning and adjusting equipment [40]. This study captured family members’ willingness to learn and participate in patient care. Such a desire to assist the patient, who is their loved one, stems from the kinship and relationship between family members and the patient, precipitating their desire to assist the patient [41]. Also, being able to get involved in patient care reduces family members’ fear and helplessness when their loved ones are critically ill. Family members’ involvement in patient care during the ICU stay also provides an opportunity for them to acquire relevant skills that enables them to care for the patient after discharge [26]. Nonetheless, such involvement necessitates additional attention and careful supervision from healthcare providers, which adds to their workload and may cause task delays [41, 42]. Concerns about poor quality patient care, accidental extubation, and failure to adhere to infectious control measures have also been raised when family members are involved in patient care [43]. Hence, a clear policy is formed in the local ICU to direct the selection of patients and family members, the level of their involvement in patient care, and to offer ongoing supervision while they are involved in patient care.

In terms of the psychological needs of the patients, family members acknowledged the ICU personnel as vital individuals for providing psychological support to the patients. This involves requesting that ICU personnel selectively share information with patients to offer hope to patients. Typically, the Asian family culture wishes to shield their loved ones from unpleasant news. However, this request violates the medical ethics of patient autonomy [44]. Therefore, the ICU personnel has to be trained with strong communication and negotiation skills when challenged with a need for selective non-disclosure [45]. Apart from that, patients’ need of religious support and counselling have also been articulated in this study. Spiritual distress is common among ICU patients, and thus religious support (such as praying with the patient, discussing religious topics, and fostering religious growth) is regarded as a source of encouragement and hope [46]. Meanwhile, psychological support (such as counselling, stress management, and coping strategies) provided during ICU stay has been shown to reduce post-traumatic stress disorder and the need for psychiatric medication among patients [47]. In the local ICU, spiritual support is always offered to the family based on the patients’ religious beliefs. Such effort in supporting patients’ religious and counselling needs is thus to be applauded and should be more proactively offered to those who are in need, although identifying spiritual needs is rather difficult.

The unmet physical need highlighted by the ICU patients would be an unpleasant experience during bed-bath with cold water late at night. This finding is similar to a study in Istanbul that found bed-bath was prevalent between midnight and five o’clock in the morning [48]. This practice could be due to a less busy schedule during midnight for the nurses to conduct bed-baths. Aside from the timing of bed-baths, the water temperature should be ideal. It is recommended that bed-bathing timing be based on individual patient preference [49], with the water temperature set at 40 to 42.5^o^C [50]. Though bed-bath may cause some discomfort to patients, it is vital in preserving patients’ hygiene and improving health outcomes [51].

Patients’ social needs, including seeing their family members [17], cannot be met due to the restricted visiting hours. Permission for flexible visiting hours to accommodate family members who have other commitments or different working schedules can facilitate family involvement in patient care. However, this has to be carefully considered taking into consideration the consequences on nursing care and disturbance to other patients [52]. Developing defined yet flexible visiting policies by tailoring visiting hours based on the needs of patients, families, and healthcare personnel may be more feasible and acceptable to all [53]. Currently, extended visiting hours in the ICU have been offered to family members of selected patients, particularly patients requiring long-term care. The provision of flexible visiting hours allows family members to engage in patient care with the assistance of the nurses.

Intolerable cold environments emerged as a negative experience for ICU patients when they were asked about the need for clinical spaces in the ICU. As part of the infection control recommendations, the Centers for Disease Control and Prevention (CDC) recommends that hospital wards maintain a temperature of 21-24^o^C [54]. However, the cold environment may not be well tolerated by patients [55]. To strike a balance between CDC recommendations and patients’ comfort, patients could be reassured and given extra blankets to assist them to cope with the cold environment in the ICU.

In the aspect of non-clinical space, family members expressed the need for additional waiting chairs to be placed outside the ICU. Driving this need is the desire of families for reassurance and to be in close proximity with their loved ones during the critical phase [11, 56]. In the United States, family members desired a comfortable waiting area where they could find solace in the company of other relatives of critically ill patients [57]. Unfortunately, at the ICU where the study was conducted, there was no designated ICU waiting room for family members. A hospital waiting-lounge facility is available within a five-minute walking distance from the ICU, but not all of the family members were aware of this facility [58]. They should be informed of the availability of the facility during the initial meeting with the ICU personnel.

The need for functioning call bells was raised by the participants, especially for patients who were placed in the isolated unit in the ICU. In case of an emergency, this medical device facilitates communication and connectivity of patients with healthcare providers. Patients felt safer knowing that they could reach healthcare providers for care or assistance when needed [59, 60]. It is important for the ICU personnel to monitor the function of the call bell and to set up a call bell response system. However, due to structural limitations in this ICU, functional call bells were unable to be installed, and the team was continually exploring alternatives.

Consistent with the reports of other studies, some of the devices in the ICU emit sounds and alarms, which are a source of distress for the patients [55, 61, 62]. The alarms may trigger anxiety in patients, which, when combined with the patients’ lack of familiarity with the meaning of the sounds and alarms, causes them to perceive them as a threat [63, 64]. This situation can be improved by minimizing the effects of the alarms by providing earplugs or setting “quiet times” [65, 66]. Practicing light down and reducing alarms to create “quiet times” at night have been implemented in this ICU.

### Strengths, limitations and recommendations

This research was undertaken just before the COVID-19 epidemic. The pandemic resulted in policy adjustments, including modifications to the visiting policy. Therefore, the data collection was stopped at the sixth participant when the Malaysian government declared a “Movement Controlled Order” on March 18, 2020, in response to the health emergency. Nonetheless, data from all six IDIs revolved around the same themes and no new themes emerged, data for the themes is thus saturated. Additionally, this study provides a baseline understanding of the needs and experiences of both critically ill patients and their families in Malaysia. Future research should focus on the disparities between the needs and experiences of critically ill patients and family members before and after the pandemic, and if the policy changes implemented during the COVID-19 pandemic affected their needs and experiences.

## Conclusion

This study provides a comprehensive look at the needs and experiences of critically ill patients and their families. Some of the concerns are acknowledged as having no immediate solution. The study’s findings, on the other hand, would aid ICU professionals in recognising and communicating the needs of patients and families in order to foster mutual understanding. Addressing the challenges outlined in this study could provide insights into organisational and systemic reforms to humanise ICU care.

## Data Availability

The datasets used and/or analysed during the current study are available from the corresponding author upon reasonable request.

## Abbreviation

ICU: Intensive Care Unit
